# Habituation learning: insights from zebrafish larvae

**DOI:** 10.3389/fnmol.2025.1697688

**Published:** 2025-11-14

**Authors:** Laura Köcher, Dominik Straumann

**Affiliations:** 1 University of Zurich, Zurich, Switzerland; 2 University Hospital Zurich, Zurich, Switzerland

**Keywords:** zebrafish, neuromodulation, behavior, learning, neuronal circuits, neurological and neurodevelopmental diseases, habituation, sensory filtering

## Abstract

Habituation is evolutionary conserved and often considered as one of the simplest forms of learning, however, the underlying mechanisms are highly complex. Extensive research has been conducted over the last few decades to understand the mechanisms of habituation in vertebrate and invertebrate species. Zebrafish (*Danio rerio*) has emerged as a crucial model for exploring the underlying mechanisms of habituation. Due to the possibility for genetic manipulations and non-invasive visualization of neuronal activity across the entire larval brain and genetically encoded fluorescent sensors allowing the detection of different neurotransmitters linked to behavioral processes, larval zebrafish provides a great vertebrate model to investigate habituation learning. In our review, we summarize recent insights into habituation learning as well as habituation deficits under neuropathological conditions gained from zebrafish larvae.

## Introduction

For an animal, enhancing its selective attention to noteworthy environmental features allows the conservation of energy while remaining attentive. This sensory filtering requires rapid processing of incoming information and engages various neural mechanisms (Ramaswami, 2014). Habituation is defined as the ability to filter out irrelevant stimuli from important stimuli by suppressing responses to repetitive non-salient stimuli, resulting in a progressive decline in response frequency and/or magnitude (Rankin et al., 2009). Habituation is the simplest form of non-associative learning and is evolutionarily conserved across vertebrates (Carey et al., 1998; Halberstadt and Geyer, 2009; Wolman et al., 2011). As habituation allows an animal to filter out irrelevant stimuli from important stimuli, habituation learning is believed to be a prerequisite for more complex types of learning (Rankin et al., 2009).

Learning is the biological process of acquiring new knowledge, and memory is the process of reconstructing this acquired knowledge over time (Kandel et al., 2014). Non-associative learning refers to the process in which an animal's response changes toward a stimulus in absence of any association with other stimuli that would induce such change. The two major form of non-associative learning are habituation and sensitization (Ioannou and Anastassiou-Hadjicharalambous, 2021). Over the last few decades, extensive research has been conducted to understand the mechanisms of habituation in vertebrates and invertebrates (Thompson, 2009). The description of the gill-withdrawal reflex in *Aplysia* by Pinsker et al. represented a major step in neuroscience (Pinsker et al., 1970). Owing to the relative simplicity of the nervous system and accessibility to intracellular electrophysiological recordings of *Aplysia*, it was possible to dissect the cellular mechanisms that alter synaptic properties within this circuit and habituate the withdrawal reflex (Castellucci and Kandel, 1974). These studies revealed that habituation results from excitatory synaptic transmission (Castellucci and Kandel, 1974; Cohen et al., 1997). However, although habituation learning has been well studied at the behavioral level only little is known about the neuronal mechanisms underlying habituation.

In larval zebrafish (*Danio rerio*), repetitive presentation of visual, acoustic, or tactile stimuli can induce the formation of habituation memory (Wolman et al., 2011; Burgess and Granato, 2007a; Randlett et al., 2019). An acoustic stimulus triggers a rapid escape maneuver of the larvae, termed the C-bend, which is initiated by the Mauthner cells (M-cells) located in the hindbrain (Burgess and Granato, 2007b; Korn and Faber, 2005). In response to a sudden decrease in illumination, the so-called dark-flash, zebrafish larvae respond with an escape maneuver characterized by an O-bend of their body (Burgess and Granato, 2007a), which is not mediated by the M-cells (Wolman et al., 2011; Burgess and Granato, 2007b). The neuronal pathways and molecular mechanisms that induce these defensive escape responses and which have been extensively investigated in zebrafish larvae are reviewed elsewhere (Corradi and Filosa, 2021; Hamling and Schoppik, 2018; Hale et al., 2016; Wolman and Granato, 2012; Portugues and Engert, 2009; Korn and Faber, 2005; Faber et al., 1991; Eaton et al., 1991).

Habituation can be quantified as a decrease in the magnitude of the startle response or as a binary reduction in the probability of executing a startle response (Rankin et al., 2009). Several parameters, such as stimulus intensity, frequency, and number of stimuli, determine the strength and speed of habituation (Rankin et al., 2009; Thompson and Spencer, 1966). Like other forms of plasticity, habituation exists in at least two mechanistically distinct forms: transient short-term habituation and protein synthesis-dependent long-term habituation (Rankin et al., 2009; Wolman et al., 2011). Both escape behaviors, the C-bend and the O-bend in response to acoustic stimuli or dark-flash stimuli, respectively, exhibit short-term and long-term habituation (Roberts et al., 2011; Wolman et al., 2011; Roberts et al., 2016).

Here, we review the aspects of investigating habituation learning in zebrafish larvae at the molecular, circuit, and behavioral level to gain an understanding of the underlying mechanisms that drive habituation toward different stimuli (see Table 1). Furthermore, we summarize recent insights gained from studying habituation deficits in neuropathological conditions.

**Table 1:** Overview of identified genes, hormones, neurotransmitters and receptors involved in regulating habituation toward acoustic, dark-flash, and/or looming stimuli in zebrafish larvae and their course of action.

**Regulator**	**Acoustic**	**Dark-flash**	**Loom**	**References**
*pappaa*	• Promotes short-term habituation through IGF1R/PI3K/Akt signaling • Metalloprotease activity is required to promote habituation • Expressed in the M-cells and clusters of neighboring hindbrain interneurons • Reduced short-term habituation and lowered acoustic startle threshold co-segregate in *pappaa* mutants • Promotes short-term habituation independent of NMDA-R and glycine receptor • Promotes short-term habituation by antagonizing D2 dopamine receptor • *pappaa* mutants display activity within the preoptic dopaminergic neurons during short-term habituation	• *pappaa* mutant larvae do not perform O-bends toward dark-flash stimuli, thus habituation toward dark-flash cannot be evaluated		Wolman et al., 2015; Nelson et al., 2023
*pcxa*	• Required for short-term habituation • Reduced short-term habituation without affecting of acoustic sensitivity in *pcxa* mutants			Wolman et al., 2015
*nf1*	• Promotes short-term habituation	• Promotes long-term habituation through the Ras/MAPK/PI3K signaling pathway • Promotes short-term habituation through cAMP signaling • Modulates habituation of the O-bend latency, while it does not affect other dark-flash response components		Wolman et al., 2014; Randlett et al., 2019
*hip14*	• Regulates synaptic depression during short-term habituation by regulating the location of Kv1.1 at the presynaptic sites of the M-cell • Localization of Kv1.1 is mediated through palmitoyltransferase activity of Hip14 • Acts independent of PAPP-AA/IGF1R and NF1 short-term habituation pathways • Cooperates with dopamine and NMDA receptor signaling to promote short-term habituation • *hip14* mutants display hyperexcitability across forebrain and hindbrain during short-term habituation	• Promotes short-term habituation toward dark-flash stimuli		Nelson et al., 2020; Wolman et al., 2014; Nelson et al., 2023
*ap2s1*	• Acts within heterotetrameric AP2 complex, and not independently, to promote short-term habituation • Promotes short-term habituation by antagonizing dopamine receptor signaling • *ap2s1* mutants display higher activity in the telencephalon and lower activity within the rhombencephalon compared to siblings during short-term habituation • *ap2s1* mutants display higher activity within dopaminergic neurons in olfactory bulb regions under short-term habituation	• Suppresses short-term habituation toward dark-flash stimuli		Zúñiga Mouret et al., 2024; Nelson et al., 2023
*cacna2d3*	• Reduced short-term habituation and lowered acoustic startle threshold co-segregate in *cacna2d* mutants • Acts independent of NMDA, dopamine, and glycine receptor signaling to regulate short-term habituation			Santistevan et al., 2022; Nelson et al., 2023
*cdh16*	• Promotes short-term habituation by regulating calcium homeostasis • Regulates whole-body calcium by suppressing the expression of *stc1l*, which represses environmental calcium uptake through suppression of Papp-aa • Reduced short-term habituation and lowered acoustic startle threshold co-segregate in *cdh16* mutants	• Short-term habituation toward dark-flash stimuli is not affected in *cdh16* mutants		Schloss et al., 2025; Hwang, 2009; Li et al., 2021
*kif2a*		• Deficient long-term habituation in *kif2a* mutants		Partoens et al., 2021
Melatonin		• Promote long-term habituation toward dark-flash stimuli • Primarily affects habituation of the O-bend response probability		Lamiré et al., 2023
Ethinyl estradiol	• Does not affect acoustic startle response habituation	• Potentiates long-term habituation toward dark-flash stimuli • Does not mediate its promoting effects on dark-flash habituation through the estrogen receptors (Esr1, Esr2) or the G protein-coupled estrogen receptor 1 (Gper1) • *esr1, ers2* and *gper1* mutant larvae show increased long-term habituation toward dark-flash stimuli		Lamiré et al., 2023; Hsiao et al., 2025
Serotonin	• Reduces short-term habituation through 5-HT2 serotonin receptor • Serotonergic dorsal raphe nucleus (DRN) neurons project axons near the M-cell dendrites • Sound-evoked activity of DRN neurons suppresses short-term habituation • Reduction in DRN serotonin increases short-term habituation • Activity decrease of serotonergic DRN neurons is proportional to behavioral short-term habituation			Wolman et al., 2011; Pantoja et al., 2016
Dopamine	• Promotes short-term habituation through D2 and D3 dopamine receptors • Increased short-term habituation associated with increased spontaneous activity in caudal hypothalamus dopaminergic neurons • D2 receptor inhibition results in hindbrain hyperactivation and suppression of forebrain and diencephalic activity during short-term habituation • Cooperates with Hip14 and NMDA-R signaling to drive short-term habituation	• D2 receptor antagonism increases habituation for O-bend latency and decreases habituation for O-bend amplitude	• Individual-individual differences in short-term acoustic startle habituation do not co-segregate with differences in visual short-term habituation	Wolman et al., 2011; Pantoja et al., 2016; Randlett et al., 2019; Pantoja et al., 2020; Nelson et al., 2023
GABA	• GABA_A_ receptor antagonism reduces short-term acoustic startle habituation • GABAc receptor agonism promotes short-term acoustic startle habituation	• GABA_A_ and/or GABA_C_ receptor antagonism suppresses dark-flash habituation • GABAergic neurons display short burst of activity toward stimulus onset	• Some dimming-responsive neurons are GABAergic • Neurons of the dimming-responsive cluster which increase their activity drive habituation to dark looming stimuli through GABAergic inhibitory influence onto loom-response neurons	Wolman et al., 2011; Lamiré et al., 2023; Fotowat and Engert, 2023
Glycine	• Short-term habituation is glycine-dependent • Glycinergic inhibition dampens the responsiveness of M-cells during short-term habituation • Glycine receptor inhibition produces widespread hyperactivation during short-term habituation			Marsden and Granato, 2015; Nelson et al., 2023
NMDA-R	• Requisite for short-term habituation toward acoustic stimuli • Short-term habituation is NMDA-R-dependent at different frequency ranges • NMDA-R-dependent depression of glutamate release at the axon initial segment of the M-cell • Spinal fiber neurons alter short-term habituation by NMDA-R-dependent activity at the axon initial segment of the M-cell • Inhibition suppresses activity within the subpallium, habenula, and hypothalamus during short-term habituation • Cooperates with dopamine receptor signaling and Hip14 to promote short-term habituation			Roberts et al., 2011; Wolman et al., 2011; Marsden and Granato, 2015; Bátora et al., 2021; Nelson et al., 2023

## Molecular mechanisms of habituation

Over the past decade, significant progress has been made toward understanding the mechanisms of habituation learning in *C. elegans* and *Drosophila* at the molecular level, including different key regulators, their course of action and degree of interaction (Crawley et al., 2017; Ardiel et al., 2016; Eddison et al., 2012; Wolf et al., 2007; Rankin and Wicks, 2000). Given the increased complexity of the vertebrate nervous system, it remains still largely open whether additional molecular regulators exist in comparison to invertebrates that drive habituation in vertebrates.

By utilizing a genome-wide approach, several genes have been identified that are linked to habituation deficits in zebrafish, including the pregnancy associated plasma protein-aa (*pappaa*), pyruvate carboxylase a (*pcxa*) (Wolman et al., 2015), huntingtin interacting protein 14 (*hip14*), potassium voltage-gated channel member 1a (*kcna1a*) which encodes the shaker-like voltage-gated K+ channel subunit Kv1.1 (Nelson et al., 2020), the calcium voltage-gated channel auxiliary subunit alpha-2/delta-3 (*cacna2d3*) (Santistevan et al., 2022) and *ap2s1* which encodes Adapter Protein 2 subunit σ (AP2σ) (Zúñiga Mouret et al., 2024). These genome-wide approach present a step toward describing the molecular players involved in habituation in the vertebrate system.

*pappaa* is expressed in the M-cells and several clusters of neighboring hindbrain interneurons known to modulate M-cell activation (Wolman et al., 2015) and encodes an extracellular metalloprotease known to increase insulin-like growth factor (IGF) bioavailability to bind to its receptor by cleaving insulin-like growth factor binding protein (Conover et al., 2004). *pappaa* mutant larvae display habituation deficits toward acoustic stimuli. Pharmacological activation of the two IGF1R signaling downstream effectors PI3-kinase (PI3K) or Akt, respectively, improves acoustic startle response habituation in *pappaa* mutant larvae. Accordingly, PAPP-A regulates acoustic startle response habituation through IGF signaling and its metalloprotease activity is required for this (Wolman et al., 2015). Deploying *nf1* mutant larvae to investigate pathways that control neurofibromin-dependent habituation Wolman et al. found that *nf1* mutant larvae exhibit reduced long-term habituation of the dark-flash response. Inhibition of the Ras effectors MAPK and PI3K improved long-term habituation in *nf1* mutant supporting that neurofibromin acts through the Ras/MAPK/PI3K signaling pathway to regulate long-term memory. Besides affecting long-term habituation toward dark-flash stimuli, *nf1* deficiency also reduces short-term habituation toward acoustic and dark-flash stimuli. Enhancing cAMP signaling has been shown to be sufficient to improve short-term habituation of the dark-flash response in *nf1* mutant larvae suggesting that cAMP signaling regulates habituation in a NF1-dependent manner (Wolman et al., 2014). The palmitoyltransferase Hip14 regulates synaptic depression of the M-cell lateral dendrites during acoustic startle response habituation and regulates habituation learning through the voltage-gated K+ channel subunit Kv1.1. Localization of Kv1.1 to presynaptic terminals of the M-cell is mediated through palmitoyltransferase activity of Hip14. Furthermore, pharmacological manipulations of Ras/MAPK/PI3K and cAMP signaling, respectively, suggests that Hip14 acts independent of PAPP-AA/IGF1R and NF1 habituation learning pathways (Nelson et al., 2020).

Different subunits of the AP2 complex modulate acoustic startle response habituation in a similar manner, suggesting that the AP2 acts as a heterotetramer, and subunits do not perform functions linked to acoustic startle response habituation independently. Disrupting *ap2s1*, which encodes subunit σ (AP2σ), results in deficient acoustic startle response habituation, while habituation toward dark-flash stimuli is enhanced. Thus, *ap2s1* modulates habituation in a sensory modality-specific manner. Because neuronal expression of *ap2s1* after embryonal stages is sufficient to rescue habituation deficits in *ap2s1* mutants, the AP2 complex seems dispensable for developmental wiring of circuits involved in acoustic habituation learning but rather targets membrane-associated proteins for endocytosis after developmental stages and thereby regulates habituation learning (Zúñiga Mouret et al., 2024). Furthermore, kinesin family member 2a (*kif2a*), regulating microtubule depolymerization via an ATP-driven process (Manning et al., 2007), has been shown to affect long-term habituation toward dark-flash stimuli. However, increased apoptosis and deficient neuronal cell proliferation displayed in *kif2a* mutant larvae may have contributed to deficits in long-term habituation (Partoens et al., 2021).

While most of the identified mechanisms of habituation act within brain circuits, non-autonomous regulators have been largely unexplored. More recently, Cadherin-16 (Cdh16) has been described to function via an endocrine organ to regulate calcium homeostasis and ultimately regulate sensory gating through habituation (Schloss et al., 2025). Cdh16 regulates whole-body calcium by suppressing the expression of the hormone Stanniocalcin 1l (*stc1l*). Stc1l limits proliferation of a specific class of ionocytes, specialized to promote calcium uptake from the environment, through suppression of Papp-aa (Hwang, 2009; Li et al., 2021). In absence of Cdh16, calcium uptake is severely limited and acoustic startle threshold is lowered resulting in deficient acoustic startle response habituation of the larvae. Interestingly, habituation toward dark-flash stimuli is not affected, indicating that Cdh16 regulates habituation learning through calcium homeostasis in a sensory-specific manner. Similar as for AP2σ (Zúñiga Mouret et al., 2024), Cdh16 seems to regulate habituation learning after establishment of the acoustic startle circuit (Schloss et al., 2025).

Deficits in acoustic startle response habituation frequently co-segregate with a lowered acoustic startle threshold, as in the case of *cacna2d3* (Santistevan et al., 2022)*, pappaa* (Wolman et al., 2015) or *cdh16* mutant larvae (Schloss et al., 2025). However, in *pcxa* mutant larvae reduced acoustic startle response habituation occurred independently of acoustic sensitivity (Wolman et al., 2015) and vice versa, other mutations caused acoustic hypersensitivity of the larvae without affecting acoustic startle response habituation (Marsden et al., 2018). Thus, genetic pathways that control these two behaviors seem to partially overlap, and are to some extent controlled by independent molecular pathways.

## Neuronal circuits involved in habituation

Both, short- and long-term habituation of the acoustic startle response are N-methyl-D-aspartate-receptor (NMDA-R)-depended (Roberts et al., 2011; Marsden and Granato, 2015; Roberts et al., 2016). Additionally, short-term habituation of the acoustic startle response also depends on the glycine receptor (Marsden and Granato, 2015).

The M-cell receives inputs at different sites which converge and can give rise to different behavioral outputs (Hale et al., 2016). At the axon initial segment of the M-cell glutamate release is dependent on NMDA-R activity and this glutamate release displays a NMDA-R-dependent depression. Whereas at the lateral dendrite glutamate release is not regulated by NMDA-R but instead shows a frequency-dependent depression (Bátora et al., 2021). Further, activity of the M-cell lateral dendrite determines acoustic startle probability (Marsden and Granato, 2015). Together this suggests that the axon initial segment of the M-cell might be responsible for inducing depression of synaptic activity during short-term acoustic startle response habituation, and the lateral dendrite of the M-cell serves as a baseline component for the startle threshold. While a set of spinal inhibitory neurons, CoLos, have been ruled out as a source for downstream inhibition of the M-cell during acoustic startle response habituation (Marsden and Granato, 2015), a feed-forward interneuron population has been noticed which affects habituation by modulating the axon initial segment of the M-cell (Bátora et al., 2021).

During habituation of the acoustic startle response the activity of dorsal raphe nucleus serotonergic neurons, which project their axons near the M-cell dendrites, decreases, and the amount of this decrease is proportional to behavioral habituation. Short-term habituation of the acoustic startle response differs between individual larvae; these differences are stable over developmental days and heritable. Reducing serotonin content in dorsal raphe nucleus neurons increases habituation, whereas serotonergic agonism or dorsal raphe nucleus activation reduces habituation, suggesting that inter-individual differences in serotonergic signaling in the M-cell circuit affect behavioral output (Pantoja et al., 2016). Individuals who habituated quickly to acoustic stimuli displayed increased spontaneous activity in dopaminergic caudal hypothalamic neurons. Thus, serotonin operates contrary to dopamine in regulating habituation to acoustic stimuli. Furthermore, Pantoja et al. showed that inter-individual differences in acoustic startle response habituation affect whole-brain activity patterns. Individuals with slow habituation rates displayed increased activity in the absence of any stimuli in brain regions (such as the habenula, interpeduncular nucleus, and pallium), which were activated by acoustic stimuli in fast habituating individuals (Pantoja et al., 2020).

Also, for long-term habituation of the dark-flash response several relevant pathways have been identified, including melatonin, estrogen, and GABAergic signaling. Partial antagonism of GABA_A_ and/or GABA_C_ receptors strongly suppresses dark-flash response habituation, highlighting an important role of GABAergic signaling for habituation learning. Using Ca^2+^ imaging while presenting repeated dark-flash to the larvae they further identified GABAergic neurons characterized by a short burst of activity to the stimulus onset (Lamiré et al., 2023). The involvement of GABAergic signaling suggests a model in which the potentiation of inhibition suppresses sensory-induced motor output during habituation (Ramaswami, 2014). In contrast to GABAergic signaling, melatonin and estrogen promote behavioral habituation toward dark-flash stimuli. Melatonin primarily affects habituation of the response probability by inducing a more rapid decay, while it does not strongly alter habituation of the displacement of the larvae after executing a O-bend. This suggests that melatonin modulates specific behavioral aspects during long-term habituation (Lamiré et al., 2023). Ethinyl estradiol, an estrogen receptor agonist, also promotes long-term habituation toward dark-flash stimuli, however, acoustic startle response habituation is unaffected (Lamiré et al., 2023; Hsiao et al., 2025). Moreover, estradiol does not mediate its promoting effects on dark-flash habituation through the two main classes of estrogen receptors. Notably, *esr1, esr2a*, and *gper1* mutants, deficient for one of the estrogen receptors, displayed increased habituation, indicating that estrogen receptors inhibit long-term habituation. Accordingly, it is less evident so far how specific estrogen signaling modulates long-term habituation toward dark-flash stimuli (Hsiao et al., 2025).

Thus, several regulators and neurotransmitters have been identified as contributing to habituation. To shed light on whether these molecular pathways and neurotransmitter systems act independently or interact with each other, as well as whether they act through overlapping neuronal circuits, Nelson et al. combined pharmacogenetic pathway analysis with whole-brain activity mapping and monitored acoustic startle response habituation.

Antagonizing the NMDA or dopamine receptor reduced habituation to acoustic stimuli in wild-type larvae, whereas the same treatment in *hip14* mutant animals did not further reduce habituation. Furthermore, *hip14* mutant animals and treatment with NMDA or dopamine receptor inhibitors displayed similar whole-brain activity patterns. Therefore, they suggested that Hip14 acts in a common pathway with the NMDA-R and the dopamine receptor to promote acoustic startle response habituation. In contrast, glycine-receptor inhibitor reduced habituation in both *hip14* mutant and wildtype animals, suggesting that Hip14 and glycine have independent roles in regulating acoustic startle response habituation. Finally, while in wildtype larvae, dopamine receptor inhibition impaired habituation, the same treatment restored habituation in *ap2s1* mutants. Papp-aa promotes habituation independent of NMDA and glycine receptor signaling but seems to be required to suppress dopamine signaling during habituation learning. Accordingly, Hip14 cooperates with dopamine and NMDA-R signaling to foster habituation, whereas Ap2s1 and Papp-aa fosters habituation by antagonizing dopamine signaling, proposing two opposing roles for dopaminergic neuromodulation. Additionally, in *ap2s1* and *pappaa* mutant larvae neuronal activity following presentation of repetitive acoustic stimuli was increased in regions labeling dopaminergic neurons, suggesting that Ap2s1 and Papp-aa regulate habituation by limiting endogenous dopamine signaling through modulation of activity of dopaminergic neurons.

Using the same pharmacogenetic pathway analysis, they found that *cacna2d3* and Kv1.1, respectively, act independent of NMDA, dopamine, and glycine receptor signaling to regulate habituation learning. Interestingly, in *hip14* mutants, changes in neuronal activity upon repetitive habituating acoustic stimuli occurred in several regions of the larvae brain, while in *kcna1a* mutant brains these changes were more restricted to a set of hindbrain neurons. Together with the fact that *hip14* mutants displayed more severe acoustic startle response habituation deficits than *kcna1a* mutants, supports the idea that Hip14 targets additional substrates beside Kv1.1 to modulate acoustic startle response habituation (Nelson et al., 2020, 2023). By grouping eight molecular regulators of habituation into five distinct pathways, termed modules, they found that three of the modules were functionally interconnected: module 1 (consisting of Hip14, as well as NMDA and dopamine receptor signaling), module 2 (consisting of Hip14 and Kv1.1), and module 3 (consisting of Papp-aa and Ap2s1). Module 4 (defined by Glycine signaling) and module 5 (defined by *cacna2d3*) seem to be functionally independent from each other and all other three modules, proposing that some habituation regulating pathways act in parallel (Nelson et al., 2023).

By examining neuronal activity while presenting repeated dark-flash to the larva, Lamiré et al. further identified functional groups of neurons that differed based on their rate of adaptation, stimulus response shape, and anatomical locations. Most groups of neurons attenuated their responses to repeated stimuli; however, they identified populations of neurons that did not adapt their responses to repeated stimuli. These non-adapting were distributed across brain areas, suggesting a distributed habituation learning process (Lamiré et al., 2023). Such a model of a distributed habituation learning process, in which non-habituated signals are transmitted throughout the brain, is further supported with brain-wide imaging data where distributed neurons displayed differential rates of habituation toward looming stimuli (Marquez-Legorreta et al., 2022). Marquez-Legorreta et al. showed that distinct functional categories of loom-sensitive neurons are located at characteristic locations throughout the brain. Using graph theory, they identified a visual circuit that habituates minimally toward loom stimuli, a moderately habituating population of neurons throughout the midbrain suggested to mediate the sensorimotor transformation, and a population of neurons in premotor regions located in the hindbrain and higher-order forebrain regions that represent threats (Marquez-Legorreta et al., 2022). To further investigate the neuronal substrates of the dark-looming stimulus, Fotowat and Engert performed calcium imaging in larvae in response to visual stimulation and constructed a circuit model of visually evoked escape behavior, suggesting the presence of two separate pathways. One relays visual information to the escape network in the brain stem to generate escape maneuvers, while the second one emerges to inhibit these escape responses. The second pathway is under contextual modulation, which is responsible for progressively suppressing escapes (Fotowat and Engert, 2023).

## Behavioral decomposition of habituation

Through behavioral analysis, Randlett et al. showed that habituation of the response to repetitive dark-flash stimuli involves the adjustment of multiple behavioral parameters, including the latency of the response, movement duration, and maximal bend amplitude. These different behavioral components adapt with different habituation kinetics and habituate independently of each other. They found that habituation of a few components involves dopaminergic or serotonergic signaling, some require Nf1, while others do not, and only one of the behavioral components is modulated by the circadian rhythm. Thus, habituation of different behavioral response components occurs through separate molecular mechanisms, and the modularity of behavioral habituation is based on the context of the animal. This points toward a modular model in which visual habituation emerges from multiple independent processes, each of which controls the adaptation of specific behavioral components (Randlett et al., 2019). Since most studies quantify habituation as a decrease in the magnitude of the startle response or as a binary reduction in the probability of executing a startle response, these findings highlight a relatively unexplored aspect of behavioral habituation.

Growing evidence points toward a phenotypic plasticity in learning among vertebrates (Miner et al., 2005). Larvae exposed to an enriched environment after hatching display enhanced short-term habituation learning toward acoustic stimuli at juvenile stages (Gatto et al., 2024). Further highlighting that inter-individual behavioral variability may emerge due to genetic (Pantoja et al., 2016, 2020) and environmental components (Gatto et al., 2024).

## Habituation impairment in neurological disease

Impairments in sensory filtering are common in many neurological disorders, and deficiencies in habituation have been associated with several neurodevelopmental and neurodegenerative diseases and traumatic brain injuries (Perry et al., 2007; Chen et al., 2016; Papesh et al., 2019). Mutations in several genes encoding key regulators of habituation identified in zebrafish larvae are also associated with cognitive deficits and learning disabilities in humans (Wolman et al., 2014, 2015; Nelson et al., 2020; Santistevan et al., 2022; Zúñiga Mouret et al., 2024).

Mutations in AP2S1 alleles have been associated with ASD and ADHD in humans and may result in learning disabilities and cognitive deficits. Disrupting *ap2s1* in zebrafish larvae results in severe acoustic startle response habituation deficits, replicating some of the behavioral phenotypes in humans with ASD, such as increased sensitivity and reduced habituation of behavioral responses to acoustic stimuli (Zúñiga Mouret et al., 2024; Satterstrom et al., 2020; Hannan et al., 2015).

Nonsense mutation in the highly conserved *fmr1* gene, the silencing of which causes Fragile X syndrome (FXS) in humans, leads to decreased habituation toward visual stimuli in larvae, replicating a behavioral phenotype in human patients with FXS (Marquez-Legorreta et al., 2022; Constantin et al., 2020; Ethridge et al., 2016). Moreover, *fmr1* mutant larvae showed increased network correlations together with greater transmission from sensory to premotor regions, which suggests a mechanism for slower sensorimotor learning in patients with FXS (Marquez-Legorreta et al., 2022).

Neurofibromatosis type 1 (NF1) is associated, in addition to a broad range of clinical characteristics, with learning disabilities, cognitive deficits, and attention deficits (Cichowski and Jacks, 2001; Hyman et al., 2006). Larvae deficient for *nf1* display short- and long-term habituation learning deficits with characteristics reminiscent in human NF1 patients. Furthermore, habituation deficits caused by genetic loss of *nf1* in zebrafish larvae are reversible by targeting NF1 downstream signaling pathways supporting the investigation of therapeutical targets in the treatment of behavioral dysfunction in NF1 patients (Wolman et al., 2014).

Further, zebrafish larvae have been also used to study mutations reported in lissencephaly, microcephaly, and drug-resistant epilepsy by looking at habituation learning (Partoens et al., 2021). More recently it has been shown that habituation of the acoustic startle response in larval zebrafish is impaired after a concussive impact, and that the severity of the concussive impact affects the habituation performance of the larvae (Beppi et al., 2022; Köcher et al., 2024).

## Conclusions

Although habituation is often considered one of the simplest forms of learning, its underlying mechanisms are complex. Multiple molecular regulators are involved in converging or separating neuronal pathways in different parts of the nervous system and are activated by different types of stimuli. It remains largely undetermined whether the pathways explored so far act through overlapping neuronal circuits.

A challenge in neuroscience is to address the link between brain activity and specific behaviors. Larval zebrafish (*Danio rerio*) provide a great vertebrate model to address this aspect due to the possibility for high-throughput behavioral measurements, genetic manipulations (Orger and de Polavieja, 2017) and non-invasive visualization of neuronal activity across the entire larval brain at micrometer resolution, which remains unfeasible in other vertebrate species (Ahrens et al., 2013; Migault et al., 2018). Moreover, genetically encoded fluorescent sensors allow the detection of different neurotransmitters linked to behavioral processes (Sun et al., 2018; Day-Cooney et al., 2023).

It would be interesting to gain a more comprehensive understanding of the network process during habituation learning by determining neurotransmitter subtypes and assessing synaptic relationships between neurons. Performing calcium imaging concurrent with co-labeling neurons with transgenic markers for neurotransmitter subtypes, as well as optogenetic manipulation at specific parts of the circuit, may provide greater insight into these network processes during habituation. Behavioral analysis combined with calcium imaging approaches may also provide more in-depth insights into the brain activity underlying changes in response strategies. Behavioral sequences can often be constructed using simple rules that connect sensory input with motor output, also referred to as behavioral algorithms (Marques et al., 2018; Johnson et al., 2020). Thus, modeling techniques may help uncover the basic control principles underlying behavior and provide further insight into the basic building blocks of habituation learning toward different sensory stimuli.

Additionally, it would be interesting to examine further genes associated with neurodevelopmental diseases in zebrafish larvae to obtain a better understanding of the genetic complexity and phenotypic diversity of these diseases in the future. Investigating the underlying genetic and cellular mechanisms that regulate habituation learning may provide insights into the identification of potential therapeutic targets.
